# Impact of acute renal failure on plasmatic levels of cleaved endocan

**DOI:** 10.1186/s13054-019-2349-1

**Published:** 2019-02-19

**Authors:** Alexandre Gaudet, Erika Parmentier, Nathalie De Freitas Caires, Lucie Portier, Sylvain Dubucquoi, Julien Poissy, Thibault Duburcq, Maxence Hureau, Philippe Lassalle, Daniel Mathieu

**Affiliations:** 1 Univ. Lille, U1019 – UMR 8204 – CIIL – Center for Infection and Immunity of Lille, F-59000 Lille, France; 2 CNRS, UMR 8204, F-59000 Lille, France; 3 INSERM, U1019, F-59000 Lille, France; 4 CHU Lille, Pôle de Réanimation, Hôpital Roger Salengro, F-59000 Lille, France; 5 Lunginnov, 1 rue du Pr Calmette, F-59000 Lille, France; 6 CHU Lille, Institut d’Immunologie, Centre de Biologie Pathologie Génétique, F-59000 Lille, France; 7 Institut Pasteur de Lille, F-59000 Lille, France

Dear Editor,

Recent failures to improve the prognostic of sepsis have underlined the need for a better phenotypical description of septic subjects. In this view, endocan, an endothelial proteoglycan secreted under proinflammatory conditions, has been described as a useful biomarker to early identify patients at higher risk of poor outcomes during the time course of sepsis [1]. More recently, a major catabolite of endocan, p14, has been observed at high plasmatic levels in a series of septic subjects, paving the way for a more accurate prediction of poor outcomes in such patients. However, major variations of p14 were observed between patients in this series, with unknown clinical significance [2]. Furthermore, it is currently unknown whether p14 could undergo renal elimination.

We performed a post hoc analysis based on the data from a previously published prospective cohort of severe septic patients [3]. Ninety-nine patients underwent measurement of p14 on EDTA plasma. Plasmatic endocan cleavage ratio (ECR) was calculated as plasma p14/(endocan + p14) ratio (endocan and p14 being expressed in pmol/mL) on baseline and 24 h, 48 h, and 72 h following ICU admission. Baseline characteristics of patients are exposed in Additional file 1.

In this cohort, ECR on enrolment was correlated with baseline SAPS 2 (*ρ* = 0.36, 95% CI (0.17–0.53); *p* <  10^− 3^) and SOFA (*ρ* = 0.21 (0–0.39); *p* = 0.04). Renal SOFA was the only component of SOFA score associated with higher ECR, with median [IQR] baseline ECR observed at 0.38 [0.29–0.61] in patients with baseline renal SOFA > 2 vs 0.28 [0.19–0.36] in patients with baseline renal SOFA ≤ 2 (*p* <  10^− 3^) (Table 1). Over 72 h, patients with a baseline renal SOFA at 4 had higher median plasmatic ECR than those with baseline renal SOFA < 4 (*p* <  10^− 3^) (Fig. 1).

**Table 1:** Endocan cleavage ratio (ECR) according to patients’ characteristics

Variables	Absent	Present	Spearman *ρ* (95% CI)	*p*
Age (years)			0.18 (− 0.03–0.37)	0.08
Chronic comorbidities				
COPD	0.33 [0.21–0.42]	0.23 [0.18–0.51]		0.53
Smoker	0.33 [0.21–0.45]	0.23 [0.18–0.35]		0.15
Cardiomyopathy	0.3 [0.2–0.42]	0.33 [0.2–0.45]		0.61
Chronic kidney failure	0.31 [0.21–0.44]	0.25 [0.05–0.56]		0.7
Cirrhosis	0.31 [0.2–0.45]	0.3 [0.21–0.37]		0.63
Sepsis severity on enrolment				
Severe sepsis		0.29 [0.2–0.41]		
Septic shock		0.31 [0.2–0.45]		0.74
Site of infection on enrolment				
Soft tissues		0.3 [0.2–0.39]		
Respiratory		0.33 [0.21–0.45]		
Urinary		0.31 [0.22–0.53]		0.82
Digestive		0.31 [0.24–0.38]		
Other		0.21 [0.07–0.49]		
Biomarkers on enrolment				
CRP (mg/L)			0 (−0.21–0.2)	0.96
PCT (ng/mL)			0.06 (− 0.15–0.26)	0.58
Prognostic scores on enrolment				
SAPS 2			0.36 (0.17–0.53)	< 10^−3^
SOFA			0.21 (0–0.39)	0.04
LIPS			0.12 (−0.08–0.32)	0.22
Organ SOFA > 2 on enrolment				
Pulmonary	0.3 [0.2–0.37]	0.35 [0.24–0.48]		0.19
Renal	0.28 [0.19–0.36]	0.38 [0.29–0.61]		< 10^− 3^
Hepatic	0.32 [0.21–0.42]	0.26 [0.14–0.53]		0.75
Circulatory	0.23 [0–0.39]	0.33 [0.23–0.45]		0.12
Neurological	0.3 [0.2–0.38]	0.33 [0.24–0.48]		0.19
Hematological	0.31 [0.2–0.4]	0.27 [0.12–0.54]		0.99
Mortality				
Day 28	0.3 [0.19–0.38]	0.33 [0.23–0.48]		0.4
ICU discharge	0.3 [0.2–0.37]	0.33 [0.23–0.54]		0.24
ICU length of stay (days)			0.16 (−0.04–0.35)	0.11
Mechanical ventilation on enrolment	0.3 [0.19–0.37]	0.31 [0.23–0.47]		0.33

ECR are presented as median [IQR] values according to presence or absence of categorical variables. A Mann-Whitney test was used for comparison between two groups. A Kruskal-Wallis test was used for comparison between three or more groups. Correlations between ECR and continuous variables are described through Spearman *ρ* (95% CI)

*COPD* chronic obstructive pulmonary disease, *SOFA* Sequential Organ Failure Assessment, *ICU* intensive care unit, *SAPS 2* Simplified Acute Physiology Score 2, *LIPS* Lung Injury Prediction Score

**Fig. 1:**
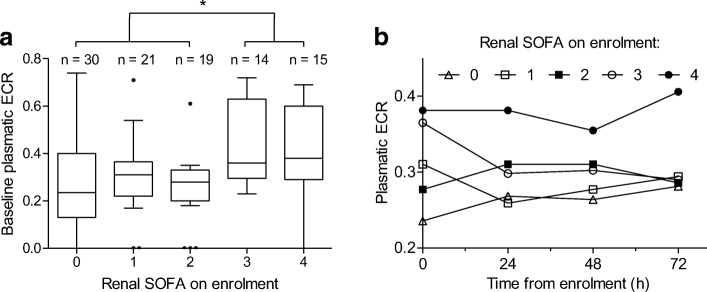
**a** Box plots of plasmatic endocan cleavage ratio (ECR) on enrolment according to baseline renal SOFA. Box plot shows the median (horizontal line) and IQR (25th–75th percentile) (box). The whiskers show the lowest data within 1.5 IQR of the lower quartile and highest data within 1.5 IQR of the upper quartile; data outside 1.5 IQR of the upper or lower quartiles are depicted with a dot. Comparison between subjects with renal SOFA > 2 vs renal SOFA ≤ 2 was performed with the Mann-Whitney test. *: *p* <  10^− 3^. **b** Kinetics of plasmatic ECR median values over 72 h following enrolment according to baseline renal SOFA

Our results suggest that circulating concentrations of p14 might be influenced by the severity of acute renal failure. Therefore, it could be proposed that, by contrast with endocan, p14 could be eliminated through glomerular filtration, thus suggesting that it should be measured in urine rather that in blood. This discrepancy might be explained by the smaller molecular weight of p14, as well as the absence of polyanionic glycanic chain on its protein core. Further explorations are needed to confirm these hypotheses.

## Additional file

Additional file 1: Cohort baseline characteristics. Continuous and categorical variables are described as median [interquartile range] and number (percentage), respectively. *COPD* chronic obstructive pulmonary disease, *SOFA* Sequential Organ Failure Assessment, *ICU* Intensive Care Unit *SAPS 2* Simplified Acute Physiology Score 2, *LIPS* Lung Injury Prediction Score (DOC 45 kb)

## Abbreviations

ECR: Endocan cleavage ratio
