# Supplementary material for: Impact of acute renal failure on plasmatic levels of cleaved endocan
Source: Crit Care. 2019 Feb 19;23:55. doi: 10.1186/s13054-019-2349-1 (PMC6381621; doi:10.1186/s13054-019-2349-1)
Supplement: Supplementary file 1 — Cohort baseline characteristics. Continuous and categorical variables are described as median [interquartile range] and number (percentage), respectively. COPD chronic obstructive pulmonary disease, SOFA Sequential Organ Failure Assessment, ICU Intensive Care Unit SAPS 2 Simplified Acute Physiology Score 2, LIPS Lung Injury Prediction Score (DOC 45 kb) [file 13054_2019_2349_MOESM1_ESM.doc]

**Additional file 1. Cohort baseline** characteristics

| **Variables** | **All patients**  **(n = 99)** |
| --- | --- |
|
| Age (years) | 63 [52 – 75] |
| Sex (male) | 59 (59%) |
| Chronic comorbidities  COPD  Smoker  Cardiomyopathy  Chronic kidney failure  Cirrhosis | 10 (10%)  23 (23%)  30 (30%)  4 (4%)  10 (10%) |
| Sepsis severity on enrolment  Severe sepsis  Septic shock | 25 (25%)  74 (75%) |
| Site of infection on enrolment  Soft tissues  Respiratory  Urinary  Digestive  Other | 39 (39%)  31 (31%)  12 (12%)  8 (8%)  9 (9%) |
| Biomarkers on enrolment  CRP (mg/L)  PCT (ng/mL) | 232 [140 – 292]  24.4 [7.8 – 70.4] |
| Prognostic scores on enrolment  SAPS 2  SOFA  LIPS | 57 [46 – 72]  10 [6 – 13]  7.5 [5.5 – 9] |
| Organ SOFA on enrolment  Pulmonary  Renal  Hepatic  Circulatory  Neurological  Haematological | 2 [1 – 2]  1 [0 – 3]  0 [0 – 1]  4 [3 – 4]  0 [0 – 4]  0 [0 – 2] |
| Mortality  Day 28  ICU discharge | 25 (25%)  25 (25%) |
| ICU length of stay (days) | 13 [7 – 21] |
| Mechanical ventilation on enrolment | 52 (52%) |
